# Intermittent Theta Burst Stimulation vs. High-Frequency Repetitive Transcranial Magnetic Stimulation in the Treatment of Methamphetamine Patients

**DOI:** 10.3389/fpsyt.2022.842947

**Published:** 2022-04-26

**Authors:** Qingming Liu, Huimeng Sun, Yitian Hu, Qiongyao Wang, Zhiyong Zhao, Da Dong, Ying Shen

**Affiliations:** ^1^Center for Brain, Mind and Education, Shaoxing University, Shaoxing, China; ^2^School of Teacher Education, Shaoxing University, Shaoxing, China; ^3^School of Psychology, Nanjing Normal University, Nanjing, China; ^4^Key Laboratory for Biomedical Engineering of Ministry of Education, Department of Biomedical Engineering, College of Biomedical Engineering & Instrument Science, Zhejiang University, Hangzhou, China; ^5^Rehabilitation Medicine Center, The First Affiliated Hospital of Nanjing Medical University, Nanjing, China

**Keywords:** methamphetamine use disorder, iTBS, rTMS, substance abuse, addiction

## Abstract

**Background and Aims:**

In this brief report, we compare the effectiveness and safety of intermittent theta burst stimulation (iTBS) and conventional 10 Hz repetitive transcranial magnetic stimulation (rTMS) in patients with methamphetamine use disorder (MAUD). Our study suggests that iTBS would also reduce drug craving in patients with MAUD just as the 10 Hz; thus, there may be no difference in treatment effects between these two methods.

**Methods:**

In total twenty male methamphetamine (MA) addicts were randomly assigned to iTBS (*n* = 10) or 10 Hz (*n* = 10) groups for 12 treatments. Cue-evoked cravings, anxiety, depression, and withdrawal symptoms were measured at baseline before the first treatment, and post-tests after days 10, 15, and 20.

**Results:**

The results showed that iTBS and 10 Hz treatment had similar effectiveness in reducing cue-induced craving in male addicts for MA. Both 10 Hz and iTBS improved withdrawal symptoms of patients with MAUD.

**Conclusions:**

Intermittent theta burst stimulation may be similar in effectiveness as 10 Hz in treating patients with MAUD. The clinical usefulness of rTMS could be improved substantially because of the increase in its capacity, cost, and accessibility. Importantly, the effectiveness of rTMS in the treatment of patients with MAUD is not yet proven, and should be tested in the large double-blind sham-controlled studies.

## Introduction

Methamphetamine use disorder (MAUD) can cause serious social problems. It is well-accepted that patients with substance use disorder (SUD) experience high cravings and high-relapse rates. Currently, available treatments for MAUD mainly include an extension application of deep electrical stimulation therapy in the human brains based on animal optogenetic and electrical stimulation, which remain to be proven, because of the invasive nature and high price. In addition, target sites have not yet been identified. One of the most widespread addiction rehabilitation treatments in China is physical isolation, which combines physical rehabilitation and psychological counseling, but lacks targeted brain science techniques; implementation of psychological counseling requires a long period of time and extensive counselor experience. Its promotion is limited under existing conditions in China. Non-invasive brain stimulation techniques, such as repetitive transcranial magnetic stimulation (rTMS), may be a more scientifically sound option. rTMS induces sustained changes in the brain regions through high-intensity, focused pulsed magnetic fields (1, 2). Additional findings have also demonstrated the efficacy and safety of rTMS in the left dorsolateral prefrontal cortex (DLPFC) for the treatment of patients with MAUD. For instance, in patients with MAUD, previous studies found that MA abuse impairs motor cortical plasticity and function (3); rTMS can reduce cravings (4), enhance cognitive function (5), and improve withdrawal symptoms (6). These findings have been shown in women (7) and also in a larger sample of men (8).

Repetitive transcranial magnetic stimulation is approved by the U.S. Food and Drug Administration (FDA) for the treatment of major depression (9), migraine with aura (10), obsessive–compulsive disorder (11, 12), and smoking addiction (13). It is widely used in rehabilitation, psychiatry, and neurology departments in many hospitals worldwide, and has been used to treat SUD in the recent years. The FDA-approved stimulation paradigm for the treatment of depression involves high frequency (10 Hz) and 37.5 min of stimulation time (14). Excessive treatment time limits the number of treatments and increases the cost of treatment. Therefore, it is possible that reducing treatment time could improve the feasibility of rTMS and increase economic benefits. A new form of rTMS has arisen, called “theta burst stimulation” (TBS) (15, 16). Unlike 10 Hz stimulation, TBS mimics endogenous rhythms and can strengthen long-duration-enhanced conduction at synapses (16). Intermittent TBS (iTBS) is capable of delivering 600 pulses in 3 min, showing similar or stronger excitatory effects compared with conventional 10 Hz stimulation (17). Several findings have shown that iTBS is superior to sham treatment for the refractory depression (18–20). One study showed that iTBS has similar effectiveness as 10 Hz for treating patients with refractory depression (21). Several studies have used iTBS in MAUD and other forms of addiction, either alone or in combination with conventional treatments. For example, one study found that iTBS affects cocaine consumption and cocaine craving almost the same as in a 15 Hz group (22). Another study evaluated the tolerability and safety of iTBS, which reduced cocaine use in a non-treatment-seeking cohort (23). However, one study indicated that iTBS in the left DLPFC was feasible and tolerable when modulating craving and mood changes in patients with MAUD (24). Cue-induced craving, however, is often treated with inhibition protocols applied to the medial prefrontal cortex (25), but the results have been inconsistent. For instance, continuous TBS (cTBS) of the ventromedial prefrontal cortex weakened neural reactivity to drug and alcohol cues in frontostriatal circuits, but had no effect on drug-/alcohol-induced cravings (26). Therefore, in this study, we chose excitatory protocols. Our study focused on whether iTBS has similar therapeutic effect as 10 Hz in patients with MAUD. Regarding TMS intervention in patients with MAUD, if 3 min of iTBS has similar therapeutic effect as conventional 10 Hz (at least 10 min of treatment time), this greatly improves the efficiency and economic benefit of TMS use.

The effectiveness of iTBS has been confirmed in psychiatry and neurology (27–30). Meanwhile, iTBS has the advantages of short treatment time, high feasibility, and good economic benefits. Therefore, we would investigate the differences in the efficacy of iTBS and classic high-frequency rTMS protocols in patients with MAUD in this study. Then, we hypothesized that compared to the 10 Hz, iTBS has the similar therapeutic effect in patients with MAUD.

## Methods

### Participants

This study was a randomized, parallel-controlled case study. In total, twenty male MA addicts aged 24–53 years were recruited from the Gongchen Addiction Rehabilitation Center in Hangzhou, Zhejiang Province. Inclusion criteria included the use of MA (DSM-V diagnosis, positive urine test upon admission, abstinence thereafter). Exclusion criteria was other drug use, infectious disease, sleep deprivation, history of epilepsy or stroke, history of mental illness, metal implants in the brain, cochlear implants, increased intracranial pressure, traumatic brain injury, brain tumor, encephalitis, cerebrovascular disease, cerebral metabolic disease, pacemakers, history of heart disease, illiteracy, and previous rTMS treatment. Experimental procedures were approved by the Ethics Committee of Nanjing Normal University in accordance with the Declaration of Helsinki and the trial was registered in the Chinese Clinical Trial Registration Center (http://www.chictr.org.cn; no. ChiCTR17013610). All the subjects signed informed consent forms before the experiment and participated voluntarily. The 20 MA participants were randomly assigned to either 10 Hz (*n* = 10) or iTBS (*n* = 10) in a 1:1 ratio using a simple randomization procedure.

The research instruments used in this study included the Visual Analog Scale (VAS), which quantitatively assesses craving in patients with MAUD; the mood scales for assessing subjects' withdrawal symptoms were Self-Rating Anxiety Scale (SAS) (31), Self-Rating Depression Scale (SDS) (32), and Withdrawal Symptom Scale for MA Addicts (6). The aforementioned scales have good reliability and validity. The treatment apparatus used was the CCY-IA transcranial magnetic stimulation equipment (Yiruide Co., Wuhan, China). The magnetic stimulus had a biphasic waveform. The maximum stimulator output was 3.0 Tesla.

### Craving Score Assessment

Craving score is an important factor in cue-induced addictive behavior and drug relapse. In our study, we asked drug users to watch a 5 min video of MA use in a relaxed state, and then, we assessed cue-induced craving scores using the VAS, with scores ranging from 0 (not at all) to 100 (very much).

### rTMS and Experimental Design

**Figure 1:**
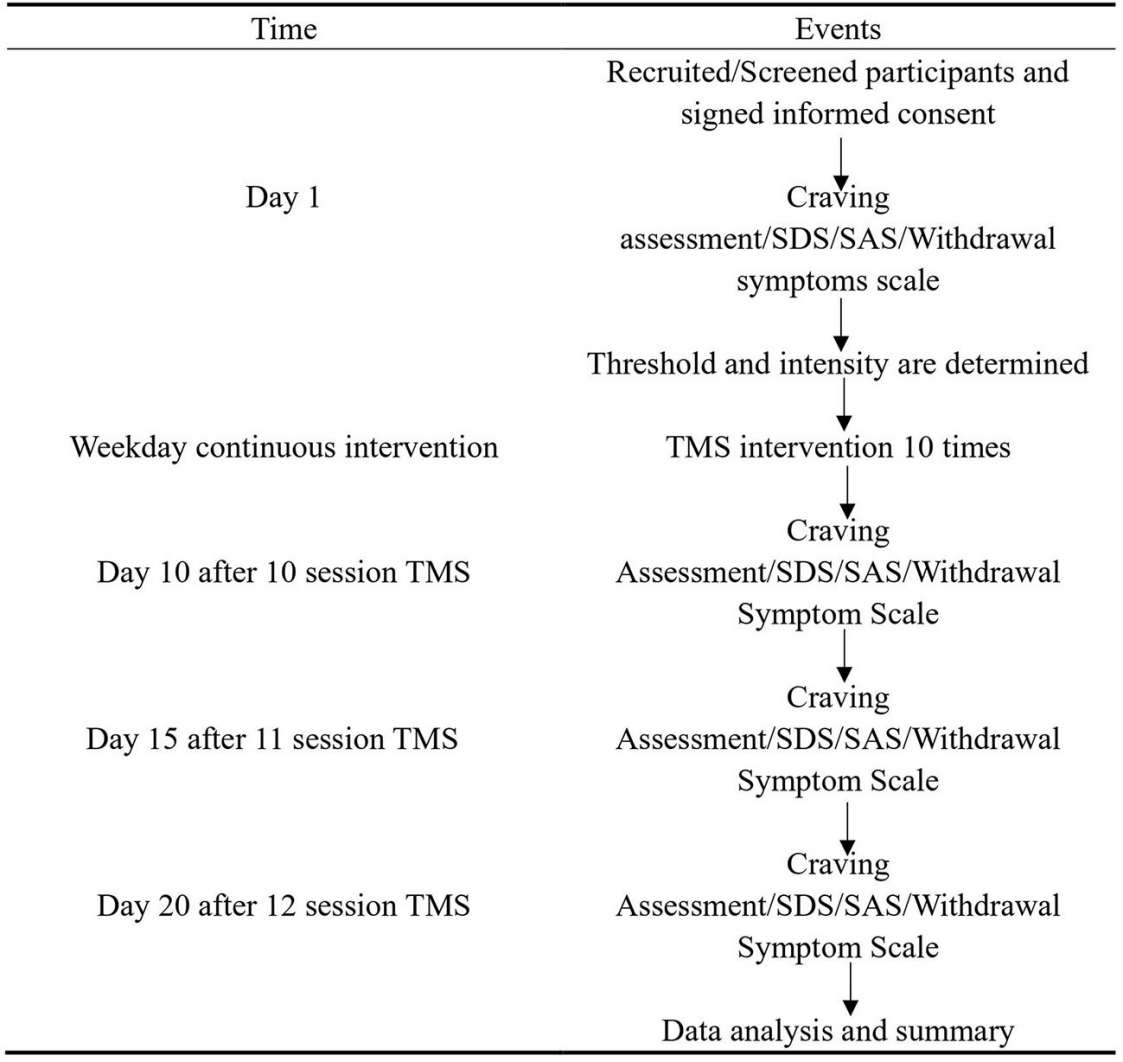
Flowchart of experimental events.

In this study, the stimulation protocol was 10 Hz or iTBS, as described in previous studies (15, 33). The parameters for 10 Hz were 5 s on and 10 s off for 10 min, 2,000 pulses. The stimulation intensity was 100% resting motor threshold (RMT). The parameters for iTBS were as follows: 50 Hz of 80% active motor threshold for three pulse trains, repeated at 5 Hz, 2 s on and 8 s off, with a total duration of 190 s, 600 pulses. The participants wore a positioning cap equipped by the Yiruide Company (10–20 EEG system). The circular coil was placed on the subject's left DLPFC at a point 5 cm anterior to the scalp position at which the motor threshold was determined (7); the stimulation was performed for 190 s or 10 min by clicking the start button on the computer screen. The treatment was performed every morning. Side effects were evaluated by asking each question according to the regulations on the instruction record sheet and scoring them (1–10, with 1 representing very mild, 5 being acceptable, and 10 being very severe). The therapist assessed the overall condition of the participant at the end of the 12 sessions. Cue-evoked cravings, anxiety, depression, and withdrawal symptoms were measured at baseline before the first treatment, and posttests after days 10, 15, and 20. The specific experimental process is illustrated in Figure 1.

### Statistical Analysis

We analyzed the data from this study using IBM Statistical Product and Service Solutions (SPSS 19.0). An independent samples *t*-test was used to compare the differences in demographic variables between the 10 Hz and iTBS groups. This study used a mixed experimental design of 2 (group: 10 Hz and iTBS) × 4 (time: pre-test, post-test, first follow-up, and second follow-up). We used two-way repeated measures ANOVA to compare the changes in craving, SAS, SDS, and MA withdrawal scores over time, between the two groups. The statistical significance threshold was set at *p* < 0.05.

## Results

### The Demographic Characteristics of the Participants

Table 1 presents the demographic characteristics of the participants, with mean ± standard error. Independent sample *t*-tests showed that there were no differences in demographic characteristics such as age [*t*_(18)_= 1.08, *p* > 0.05], years of MA intake [*t*_(18)_= 0.46, *p* > 0.05], maximum MA intake [*t*_(18)_ = 0.17, *p* > 0.05], and monthly MA intake [*t*_(18)_= 1.01, *p* > 0.05] between the 10 Hz and iTBS groups.

**Table 1:** Demographic characteristics of patients with MAUD (*M* ± *SEM*).

**Variable**	**10 Hz group (*n* = 10)**	**iTBS group (*n* = 10)**	***p***
Age (years)	38.40 ± 2.25	35.40 ± 2.66	0.37
Years of intake (years)	9.00 ± 1.18	8.50 ± 0.91	0.76
Maximum intake/per intake (g)	0.90 ± 0.19	0.72 ± 0.08	0.29
Monthly intake (g)	15.40 ± 2.79	10.70 ± 2.40	0.05

*MAUD, methamphetamine use disorder; iTBS, intermittent theta burst stimulation*.

### Effectiveness of Both 10 Hz and iTBS in Reducing Craving in Patients With MAUD

**Figure 2:**
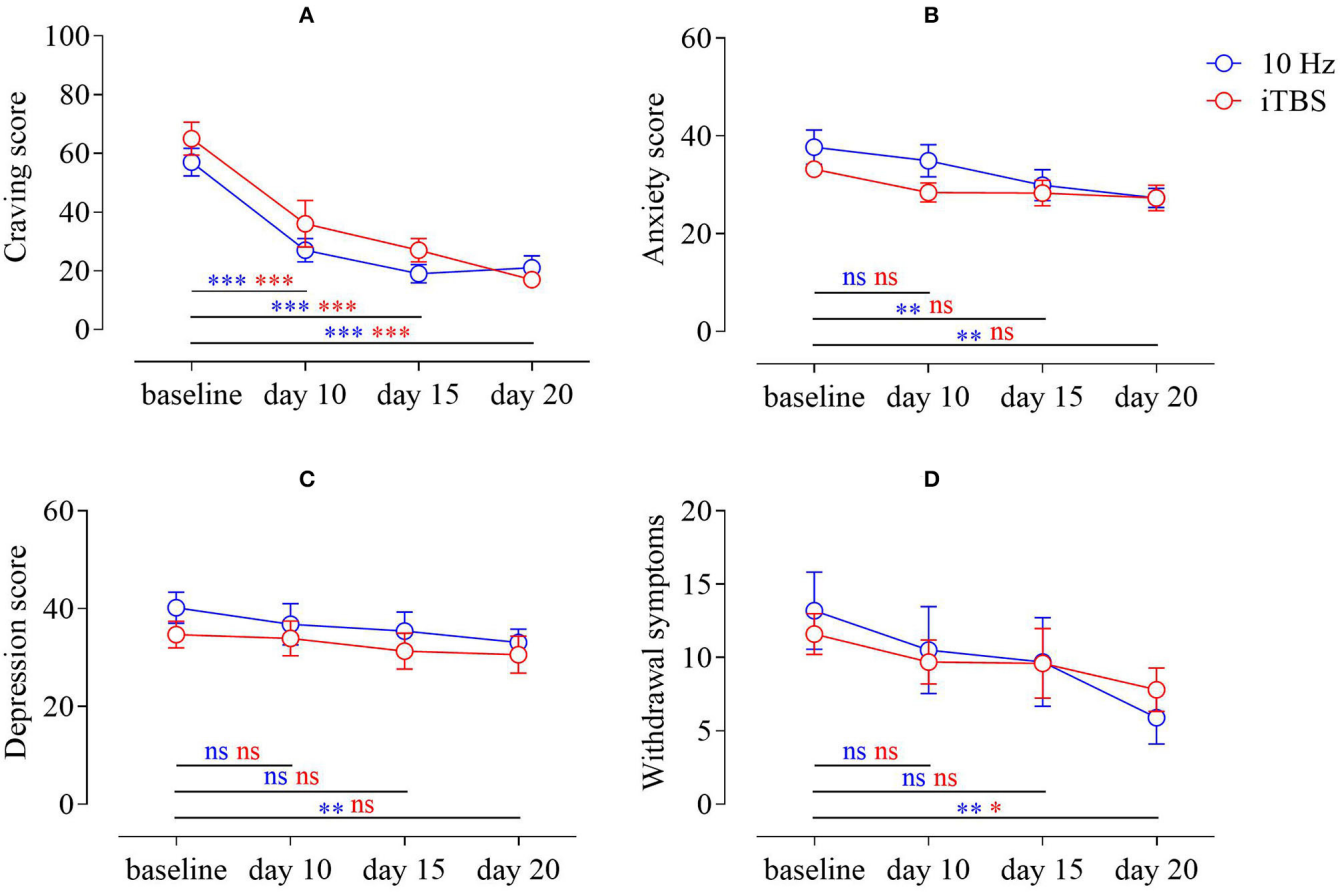
**(A)** Change in craving scores of MA addicts in the two groups; **(B)** change in anxiety scores of MA addicts in the two groups; **(C)** change in depression scores of MA addicts; **(D)** change in withdrawal symptom scores of MA addicts in both groups (MA–methamphetamine).

For craving, repeated measures ANOVA found a significant effect in time [*F*_(3,54)_ = 46.944, *p* < 0.001, $\eta_{p}^{2}$ = 0.72]. *Post-hoc* tests showed that 10 Hz reduced craving at day 10 (*M* = 27.00, *SEM* = 3.96), day 15 (*M* = 19.00, *SEM* = 3.14), and day 20 (*M* = 21.00, *SEM* = 4.07) relative to baseline (*M* = 57.00, *SEM* = 4.73). Similarly, iTBS significantly reduced craving among MA addicts on day 10 (*M* = 36.00, *SEM* = 7.92), day 15 (*M* = 27.00, *SEM* = 3.96), and day 20 (*M* = 17.00, *SEM* = 1.53) relative to baseline (*M* = 65.00, *SEM* = 5.63). The group main effect was not significant [*F*_(1,18)_ = 1.30, *p* > 0.05, $\eta_{p}^{2}$ = 0.07], and there was no interaction between time and group [*F*_(3,54)_ = 1.25, *p* > 0.05, $\eta_{p}^{2}$ = 0.07] (Figure 2A).

### SAS, SDS, MA Addict Withdrawal Symptoms Scale

For SAS, we used repeated measures ANOVA and found that there was a significant time main effect [*F*_(3,54)_ = 12.26, *p* < 0.001, $\eta_{p}^{2}$ = 0.41]. We performed *post-hoc* tests, compared with the baseline (*M* = 37.70, *SEM* = 3.50), and the results showed that 10 Hz did not improve the anxiety of MA addicts on the 10th day (*M* = 34.90, *SEM* = 3.27), but improved the anxiety on the 15th day (*M* = 29.90, *SEM* = 3.16) and day 20 (*M* = 27.30, *SEM* = 1.97) to some extent; however, relative to baseline (*M* = 33.20, *SEM* = 1.68), iTBS did not have this effect on day 10 (*M* = 28.40, *SEM* = 1.95), day 15 (*M* = 28.30, *SEM* = 2.63), and day 20 (*M* = 27.30, *SEM* = 2.61). The group main effect was not significant [*F*_(1,18)_ = 0.88, *p* > 0.05, $\eta_{p}^{2}$ = 0.05], and there was no interaction between time and group [*F*_(3,54)_ = 2.08, *p* > 0.05, $\eta_{p}^{2}$ = 0.10] (Figure 2B).

For SDS, repeated measures ANOVA found that there was a significant time main effect [*F*_(3,54)_ = 4.20, *p* < 0.05, $\eta_{p}^{2}$ = 0.19]. In *post-hoc* tests, compared with the baseline (*M* = 40.20, *SEM* = 3.17), the results showed that depression in patients with MAUD was not improved by 10 Hz on the 10th day (*M* = 36.80, *SEM* = 4.25) or the 15th day (*M* = 35.40, *SEM* = 3.90), but improved to a certain extent on the 20th day (*M* = 33.10, *SEM* = 2.73). While relative to baseline (*M* = 34.70, *SEM* = 2.72), iTBS did not have this effect on day 10 (*M* = 33.90, *SEM* = 3.60), day 15 (*M* = 31.30, *SEM* = 3.69) or day 20 (*M* = 30.60, *SEM* = 3.82). The group main effect was not significant [*F*_(1,18)_ = 0.68, *p* > 0.05, $\eta_{p}^{2}$ = 0.04], and there was no interaction between time and group [*F*_(3,54)_ = 0.32, *p* > 0.05, $\eta_{p}^{2}$ = 0.02] (Figure 2C).

In terms of withdrawal scores of patients with MAUD, repeated measures ANOVA found a significant time main effect [*F*_(3,54)_ = 9.77, *p* < 0.001, $\eta_{p}^{2}$ = 0.35]. *Post-hoc* tests were performed and compared with baseline (*M* = 13.20, *SEM* = 2.63); the results showed that the withdrawal symptoms of MA addicts at 10 Hz did not improve on day 10 (*M* = 10.50, *SEM* = 2.98) or day 15 (*M* = 9.70, *SEM* = 3.02), whereas at day 20 (*M* = 5.90, *SEM* = 1.79), there was a certain degree of improvement relative to baseline (*M* = 11.60, *SEM* = 1.38). iTBS showed no improvement on day 10 (*M* = 9.70, *SEM* = 1.51) or day 15 (*M* = 9.60, *SEM* = 2.38), but there was some improvement on day 20 (*M* = 7.80, *SEM* = 1.48). The group main effect was not significant [*F*_(1,18)_ = 0.00, *p* > 0.05, $\eta_{p}^{2}$ = 0.00], and there was no interaction between time and group [*F*_(3,54)_ = 1.06, *p* > 0.05, $\eta_{p}^{2}$ = 0.06] (Figure 2D).

In total, three participants (1 in the 10 Hz group, two in the iTBS group) reported mild dizziness or scalp pain after the first two sessions. The symptoms were relieved within 1.5 h. None of the subjects dropped out of the study due to adverse reactions. In general, both 10 Hz and iTBS reduced the cue-induced craving of male addicts for MA. In total 10 Hz or iTBS could improve withdrawal symptoms in patients with MAUD.

## Discussion

This study suggests that iTBS is similar in effectiveness as 10 Hz in reducing cravings for MA addiction. Furthermore, there was no difference between the two stimulation forms for treating patients with MAUD. Both forms of rTMS (10 Hz and iTBS) can effectively reduce cue-induced cravings in patients with MAUD, which is consistent with the conclusions of previous studies (4–7, 24). This is of great significance for improving the efficiency and economic benefits of rTMS. rTMS cannot only reduce cue-evoked cravings in patients with MAUD, but also improve anxiety and depression scores to a certain extent, and even has a positive effect on withdrawal symptoms of patients with MAUD. According to our inquiries during the study, there was no significant difference in self-reported adverse events and serious adverse events between the two groups. iTBS had a slightly higher rate of pain but did not lead to a higher dropout rate. These results indicate that 3-min iTBS can be compared with 10-min 10 Hz as an intervention for the treatment of patients with MAUD.

Although this study has certain advantages, it also has several limitations. First, the study did not design a sham group and could not properly eliminate time or placebo effects. Second, the treatment time for iTBS participants in each session was much shorter than that of the 10 Hz group, which may have led to a specific effect of time with iTBS. Third, we lacked MRI-guided neuronavigation in this study; although this method is not feasible or cost-effective for most studies conducted in addiction rehabilitation centers. As a reference, a previous study showed that in a similar experiment, BeamF3 (a heuristic method based on scalp measurements) could achieve the same stereotactic target as MRI (34). Fourth, since patients with MAUD had been in the rehabilitation center during treatment, there was a lack of urine tests to show whether the improvement in craving led to a reduction in consumption. Finally, due to the current epidemic situation, this study had a small sample size, imposing certain limitations. In future, the sample size should be expanded to further add to the significance of this study.

In conclusion, we found that iTBS may have similar therapeutic effect compared with 10 Hz in patients with MAUD. Typical iTBS treatment (including measuring motion thresholds, etc.) takes 5–10 min, while 10 Hz takes 15–20 min. Therefore, the number of patients with MAUD treated with each iTBS protocol per day can be increased by more than two-fold. In a broader sense, iTBS could have a more positive impact on the effects of enhancing treatment capacity, including improving treatment pathways, and reducing waiting times, thereby helping more patients with MAUD in addiction rehabilitation centers to benefit from the advantages of TMS and help physicians treat more patients.

## Data Availability Statement

The raw data supporting the conclusions of this article will be made available by the authors, without undue reservation.

## Ethics Statement

The study was conducted according to the guidelines of the Declaration of Helsinki, and approved by Ethics Committee of Nanjing Normal University (2017-004) and was registered in the Chinese Clinical Trial Registration Center (http://www.chictr.org.cn; no. ChiCTR17013610). The patients/participants provided their written informed consent to participate in this study.

## Author Contributions

QL: conceptualization. HS: methodology. YH and ZZ: formal analysis. QL, YS, and QW: investigation. QL and YS: data curation. QL and DD: writing, reviewing, and editing. All authors have read and agreed to the published version of the manuscript.

## Funding

This work was supported by the National Social Science Fund of China (Grant No. 20CZX015) and the National Science Foundation of China (Grant No. 81702230).

## Conflict of Interest

The authors declare that the research was conducted in the absence of any commercial or financial relationships that could be construed as a potential conflict of interest.

## Publisher's Note

All claims expressed in this article are solely those of the authors and do not necessarily represent those of their affiliated organizations, or those of the publisher, the editors and the reviewers. Any product that may be evaluated in this article, or claim that may be made by its manufacturer, is not guaranteed or endorsed by the publisher.
